# Predictors for Prehospital First-Pass Intubation Success in Germany

**DOI:** 10.3390/jcm11030887

**Published:** 2022-02-08

**Authors:** Lukas Reinert, Steffen Herdtle, Christian Hohenstein, Wilhelm Behringer, Jasmin Arrich

**Affiliations:** 1 Department of Emergency Medicine, Faculty of Medicine, Friedrich Schiller University Jena, 07747 Jena, Germany; lukas.reinert@icloud.com; 2 Department of Emergency Medicine, Hospital of Agatharied, 83734 Hausham, Germany; steffen.herdtle@khagatharied.de; 3 Department of Emergency Medicine, Zentralklinik Bad Berka, 99438 Bad Berka, Germany; christian.hohenstein@zentralklinik.de; 4 Department of Emergency Medicine, Medical University of Vienna, 1090 Wien, Austria; wilhelm.behringer@meduniwien.ac.at

**Keywords:** endotracheal intubation, prehospital emergency care, first-pass intubation success, adverse events, professional education

## Abstract

(1) Background: Endotracheal intubation in the prehospital setting is an important skill for emergency physicians, paramedics, and other members of the EMS providing airway management. Its success determines complications and patient mortality. The aim of this study was to find predictors for first-pass intubation success in the prehospital emergency setting. (2) The study was based on a retrospective analysis of a population-based registry of prehospital advanced airway management in Germany. Cases of endotracheal intubation by the emergency medical services in the cities of Tübingen and Jena between 2016 and 2019 were included. The outcome of interest was first-pass intubation success. Univariate and multivariable regression analysis were used to analyse the influence of predefined predictors, including the characteristics of patients, the intubating staff, and the clinical situation. (3) Results: A total of 308 patients were analysed. After adjustment for multiple confounders, the direct vocal cord view, a less favourable Cormack–Lehane classification, the general practitioner as medical specialty, and location and type of EMS were independent predictors for first-pass intubation success. (4) Conclusions: In physician-led emergency medical services, the laryngoscopic view, medical specialty, type of EMS, and career level are associated with FPS. The latter points towards the importance of experience and regular training in endotracheal intubation.

## 1. Introduction

Endotracheal intubation (ETI), either by direct laryngoscopy or by videolaryngoscopy, is still considered the gold standard for securing the airway [1,2,3,4,5]. It is one of the most important skills to be mastered by the members of the EMS team [2,6]. Depending on the EMS, ETI may be performed by physicians, paramedics, nurses, and airway technicians, who have undergone special training [7,8]. In the out-of-hospital setting, the procedures itself are even more challenging, as information on the patient, resources, space, and time are limited. Other life-saving procedures take place at the same time and further complicate the process of securing the airway. Failed first-pass intubation success (FPS) is associated with an increasing number of adverse events, such as desaturation and airway injury, and with higher levels of patient mortality [2,9].

Registries on emergency intubation have been established to gather data and analyse characteristics of airway management and to improve patient care [10,11]. However, the literature on factors that are associated with FPS largely depends on the type of EMS (physician-led, paramedic-led, hospital-based) and available resources (alternative methods of advanced airway management). This analysis of a population-based, two-centre registry aimed to find significant predictors for FPS for a physician-led EMS in two middle-sized cities in Germany.

## 2. Materials and Methods

This retrospective cohort study was based on a registry on prehospital advanced airway management (www.intubationsregister.de, accessed on 5 February 2022). The registry was established in April 2016 and approved by the ethics committees of the Universities of Tübingen and Jena, adhering to the general data protection regulations. The dataset of this study comprised all the reports on ETI from the Jena ambulance and emergency helicopter service between January 2016 and December 2019 and the Tübingen ambulance service from January to December 2018. Data documentation is observational only, self-reported, web-based, and available for all emergency staff in the field. Standard operating procedures are at the discretion of the physicians and paramedics involved. To be qualified for the EMS service, physicians have to complete at least two years of clinical training and additional training in prehospital emergency medicine. Paramedics may perform endotracheal intubation under the supervision of the involved emergency physician. The database comprises information on patient characteristics and the clinical situation, the presence of a cervical collar and its removal before intubation, the use of suction, intubation stylet, findings of airway examination tests, characteristics of the intubating physicians, paramedics and supervisors, medication, and adverse events. For this analysis, only cases of primary endotracheal intubation such as direct laryngoscopy and videolaryngoscopy were considered. We excluded cases where a supraglottic airway or an alternative was the first choice of advanced airway management. All potentially important variables were evaluated for a possible association with the outcome. The outcome of interest was first-pass intubation success (FPS). This was defined as the correct placement of the endotracheal tube on the first attempt. Correct tube placement was confirmed by capnography, glottis visualization, and auscultation. Failed intubation was defined by the inability to correctly place the tube, or recognition of a misplaced tube. We used the STrengthening the Reporting of OBservational studies in Epidemiology (STROBE) statement to guide the analysis and reporting of our data [12]. The self-reported data were verified by the EMS routine data documentation and underwent centralized monitoring for completeness. If available, the EMS team was contacted to obtain information on missing data.

### 2.1. Data Analysis

Continuous data were presented as mean and standard deviation, and categorical data were presented as percentages. We used univariate and multivariable logistic regression models to assess the association between first-pass intubation success and various prognostic factors: age, gender, the weight of patients, direct vocal cord view, the Cormack–Lehane (CL) classification, use of a cervical collar, intubation stylet, suction, type of emergency services (Tübingen ambulance, Jena ambulance, Jena helicopter services), the use of neuromuscular blocking agents (NMA, any NMA, rocuronium, succinylcholine, pancuronium), the type of training of the intubating emergency staff (emergency physician full-time, emergency physician part-time, emergency physician hospital-based, emergency physician trainee, other doctor on-site, paramedic full-time, paramedic part-time), the career level (senior consultant, consultant, registrar, paramedic, paramedic assistant), and the type of medical specialty (anaesthesiology, intensive care, emergency medicine, internal medicine, trauma surgery, other surgery, paediatrics, general medicine). The leading indication for intubation was stratified into cardiac arrest, trauma, and other indications. Missing variables were coded as such. Groups of variables were included as given in the dataset, only very small cell sizes were collapsed, and continuous variables were not stratified. Predictors were selected into the model by a purposeful selection of variable algorithms [13].

### 2.2. Patient and Public Involvement

Patient groups and the public were not included in the design or conduct of this study, but the dissemination plans involve publishing plain language summaries on social media and the registry’s website.

## 3. Results

Of 388 patients initially documented in the observation period, we excluded 36 intubated with a primarily placed supraglottic airway, eight where the mode of intubation was not clear, and a further 36 where it was not reported if they had FPS. The final dataset therefore comprised 308 patients. The median age was 64 years, 34% were women, and 76% had FPS. Rapid sequence intubation was the method of choice in most cases (96%). Curved blades were used almost exclusively for direct laryngoscopy, and cuffed single-lumen endotracheal tubes were used for ETI. The most used sedatives were midazolam in 41%, fentanyl in 35%, ketamine in 25%, and propofol in 16% of all cases. Table 1 shows the characteristics of patients and the emergency and the univariate analysis of possible predictors of FPS. Predictors associated with a higher FPS in the univariate analysis were a direct vocal cord view, a more favourable CL grade, the use of suction, and the type and location of the EMS (ambulance service, Tübingen, Jena).

The results of the multivariable regression analysis are summarized in Table 2. The final model consisted of 10 variables, four of which were significantly associated with FPS.

A direct vocal cord view was a strong predictor for FPS (OR 18, *p* < 0.01). In comparison to CL classification grade I, grade III (OR 0.16; *p* < 0.01) and grade IV (OR 0.04, *p* < 0.01) were associated with a lower chance of FPS. In comparison to the emergency helicopter service in Jena, FPS was less likely within the Tübingen ambulance service (OR 0.16, *p* = 0.02), but there was no significant difference between the Jena ambulance service and Jena helicopter service (OR 0.5, *p* = 0.3). There was a trend towards a higher chance for FPS for registrars in comparison to consultants (OR 3.9, *p* = 0.059). There was no difference in FPS between anaesthesiologists and other medical specialties, except for general practitioners, who had a lower chance for FPS (OR 0.07, *p* = 0.02). There was no independent association with FPS for any of the other predictors listed in Table 1 (the use of a cervical collar, suction, stylet, individual or any specific NMA).

## 4. Discussion

With this retrospective analysis, we characterized the prehospital endotracheal intubation of the ambulance services in two middle-sized German cities and found well-known but also unexpected predictors for FPS. Independent confounders associated with a lower chance of FPS were an impaired vocal cord view, a higher CL grade of III and IV, the type and location of EMS, and “general medicine” as a medical specialty. Furthermore, consultants may be less successful than registrars.

In the literature, CL classification and vocal cord view have been established as predictors for FPS in the prehospital setting, which is in line with our results [14,15,16]. There was a trend towards advanced age being a positive predictor for higher FPS. This association was even more pronounced in a sensitivity analysis when we excluded children under the age of 16 (OR 1.03, *p* = 0.01 per year). This result reflects findings from other studies on out-of-hospital endotracheal intubation [17]. There were distinct differences in the use of NMA in the EMS of the two cities, however, these were not associated with differences in the FPS rates.

In general, FPS rates of 76% may be regarded as unsatisfactory. In the literature, the FPS rate of physician-led emergency services ranges up to 90% [18,19,20], but there are also reports of FPS rates as low as 60% [21,22]. There are several arguments that claim lack of experience and training may have led to this rather low rate of successful intubations:

- In Germany, physicians are allowed to work on the EMS if they have completed clinical training of at least two years (with at least 6 months of anaesthesiology or emergency medicine training), 80 h of additional prehospital training, and 50 supervised ambulance calls. Other countries with physician-led EMS, such as France, require more extensive training, which may be a factor for a better quality of care on these EMS.
- In our data, the FPS rates were higher for the team in the emergency helicopter than the team within the ambulance services (80% versus 74%). In Germany, staff in emergency helicopters are generally more senior and experienced. The differences in FPS between the Jena helicopter and the Tübingen ambulance crew were robust to an adjustment for all the main known confounders. We do not have a definitive explanation for these differences. Potential predictors that were not documented in the registry are regular training and experience with endotracheal intubation [23,24,25].
- General practitioners, in comparison to anaesthesiologists, were less likely to intubate successfully, while there were no differences between anaesthesiologists and all other medical specialties. General practitioners in Jena and Tübingen mostly work as family doctors outside of hospitals. Studies have shown that physicians based at the emergency department have a higher chance for FPS than others [24], which may reflect the training status.
- One surprising result was that career level was not associated with FPS. On the contrary, the results indicated that registrars may even have a better chance for FPS. This again points to the possibility that intubation quality is not so much a result of career level but of the level of ongoing training and experience with ETI [25]. Studies have shown that even within residency, years of training and numbers of intubations are associated with FPS [24]. Especially in the out-of-hospital setting, constant training may be crucial for confidence in providing advanced airway management [26]. At the EMS of Tübingen and Jena, most physicians work at the hospital. As they advance in their career, their role may shift to clinical supervision. This may lead to a situation where consultants, although more experienced, are less exposed to continuous training in ETI.
- Finally, the lack of standard operating procedures, availability of videolaryngoscopy, and established regular training programs may have contributed to the low FPS rate. Low rates of drug-assisted intubations may indicate a lack of experience and training.

### 4.1. Limitations

There are some limitations of this study. The amount of data documented had to be balanced between sufficient detail of information on advanced airway management and the feasibility of data documentation in the field. The most common predictors for FPS have been documented, but the possibility for unknown confounding factors remains. Height was not included in the data documentation, and adjustment for body mass index (BMI), which may be a confounder for first-pass intubation success, was not possible. As the next best variable, the weight of patients was included, which may approximate for the influence of BMI. The use and removal of a cervical collar were documented, but not if a manual in-line stabilization was performed during intubation. Additionally, the presence of a traumatic airway obstruction and the positioning of the patient were not recorded. These would have provided more information on complicating factors, however, CL grading, at least partly, covered the presence of an airway obstruction. The maximum allowed time for intubation was not predefined. The model was adjusted for experience by the career level of physicians (registrar–consultant–senior consultant), but years of training or numbers of completed intubations could have provided a more sophisticated result. Some doctors may have more than one medical specialty. As only one answer could be selected, we assume the specialty that represented the current work was chosen. As the data entry was limited to the prehospital setting, additional outcomes, such as survival and neurologic function, were not documented.

### 4.2. Strengths

The strength of our study is the thorough data documentation, and the monitoring of self-reported data by the routine EMS database and the on-site data review. The level of detail of the data documentation was high considering that this was a prehospital clinical registry. The univariate comparison provides a general overview, while in the multivariable analysis we were able to assess the association of independent factors with FPS.

### 4.3. Generalisability

To examine external validity, the results from our full dataset were compared to the results of comparable registries in mainly unselected patients [27,28]. There was a similar age range (mean 64 years), gender ratio (34% female), and FPS rate (76%). In this database, the reasons for intubation were slightly shifted towards trauma (23% in our dataset) in comparison to the other datasets (up to 18%). Adverse events were reported in 21% of all cases, with tube displacement (11%), aspiration (6%), hypoxia (5%) and cardiac arrest (4%) being the most prevalent. Generally, the results of this manuscript reflect the findings in other studies on prehospital ETI and are applicable to physician-led EMS in middle-sized cities.

## 5. Conclusions

In this retrospective analysis of registry data on advanced airway management of physician-led emergency medical services in two middle-sized cities, we found significant factors associated with FPS for emergency endotracheal intubation. Not only the laryngoscopic view, but also the type of EMS, regular shifts at the hospital, and an earlier career level may be associated with a higher rate of FPS. These factors indicate that experience and continuous training in ETI may be one of the most important factors for FPS. Further studies should include the different aspects of physician experience, effective standard operating procedures, and training in advanced airway management. Those findings may easily be translated into improved quality of care.

## Figures and Tables

**Table 1:** Characteristics of patients, EMS providers, and endotracheal intubation. Full dataset and between groups FPS and failed FPS.

Characteristics	All	FPS	No FPS	Sig
n (%)	308	234 (76)	74 (24)	
**Patient characteristics**				
Age, mean (SD, range)	64 (20, 1–94)	64 (20, 1–94)	63 (18, 1–92)	
Female, n (%)	106 (34)	83 (35)	23 (31)	
Weight, mean (SD)	82 (21)	81 (20)	85 (24)	
**Indication for intubation**				*
Cardiac arrest	131 (43)	98 (75)	33 (25)	
Trauma	72 (23)	49 (68)	23 (32)	
Other indications	105 (34)	87 (83)	18 (17)	
Direct vocal cord view	208 (68)	191(82)	17(23)	***
**Cormack–Lehane Classification, n (%)**				***
Grade I	123 (40)	115 (93)	8 (7)	
Grade II	66 (21)	52 (79)	14 (21)	
Grade III	42 (14)	9 (21)	33 (79)	
Grade IV	11 (4)	3 (27)	8 (73)	
Not reported	66 (21)	55 (83)	11 (17)	
Cervical collar	61 (20)	43 (18)	17 (23)	
Cervical collar removed	40 (13)	29 (72)	11 (28)	
Intubation with a cervical collar	21 (7)	15 (71)	6 (29)	
Use of suction	91 (30)	58 (25)	33 (45)	***
Use of intubation stylet	292 (95)	219 (94)	73 (99)	
Videolaryngoscopy	54 (18)	40 (17)	14 (19)	
**Type of EMS**				***
Tübingen ambulance service	47 (15)	27 (57)	20 (43)	
Jena ambulance service	155 (50)	123 (79)	32 (21)	
Jena helicopter service	106 (34)	84 (79)	22 (21)	
**Intubating staff**				
**Career level, n (%)**				*
Senior consultant	73 (24)	54 (74)	19 (26)	
Consultant	114 (37)	90 (79)	24 (21)	
Registrar	84 (27)	66 (79)	18 (21)	
Paramedic	20 (6)	15 (75)	5 (25)	
Paramedic assistant	3 (1)	0	3 (100)	
Not reported	14 (5)	9 (64)	5 (36)	
**Leading medical specialty, n (%)**				
Anaesthesiology	180 (59)	137 (76)	43 (24)	
Intensive care	42 (14)	34 (81)	8 (19)	
Emergency medicine	27 (9)	21 (78)	6 (22)	
Internal medicine	16 (5)	13 (81)	3 (19)	
Trauma surgery	2 (0.7)	1 (50)	1 (50)	
Surgery, other	3 (1)	2 (67)	1 (33)	
General medicine	8 (3)	5 (63)	3 (37)	
Not reported	30 (10)	21 (70)	9 (30)	
**Medication**				
Drug-assisted intubations	179 (58)	135 (58)	44 (59)	
Use of NMA, n (%)	166 (53)	128 (77)	38 (23)	0.6
Rocuronium	95 (31)	75 (49)	20 (51)	
Succinylcholine	59 (19)	42 (71)	17 (29)	
Pancuronium	1 (0.3)	1 (100)	0	
More than one NMA	11 (4)	10 (90)	1 (10)	

FPS, first-pass intubation success; Sig, *p*-value; ***, *p* < 0.01; *, *p* < 0.1; NMA, neuromuscular blocking agent; EMS, emergency medical services.

**Table 2:** Multivariable analysis of FPS characteristics of patients, EMS providers, and endotracheal intubation. Full dataset and between groups FPS and failed FPS.

FPS	Odds Ratio	95% Confidence Interval		*p*-Value	Sig
		LL	UL		
**Patients and intubation**					
Age (per year)	1.02	1.0	1.05	0.07	*
Sex (woman)	1.42	0.56	3.621	0.464	
Weight (per kg)	1	0.98	1.017	0.73	
Vocal cord view	17.88	6.84	46.74	<0.01	***
Video laryngoscopy used	2.39	0.67	8.56	0.179	
Cormack–Lehane classification					
Grade I	1				
Grade II	0.28	0.08	1.05	0.059	*
Grade III	0.02	0.004	0.07	<0.01	***
Grade IV	0.04	0.006	0.28	<0.01	***
Not reported	0.29	0.08	1.02	0.054	*
**Location and type of emergency service**					
Jena helicopter service	1				
Jena ambulance service	0.51	0.15	1.77	0.292	
Tübingen ambulance service	0.16	0.03	0.75	0.02	**
Experience					
Senior Consultant	1				
Consultant	1.13	0.327	3.90	0.848	
Registrar	3.93	0.948	16.26	0.059	*
Paramedic	0.1	0.005	2.13	0.14	
Missing	0.39	0.044	3.41	0.393	
**Type of specialty**					
Anaesthesiology	1				
Intensive care	0.57	0.148	2.20	0.415	
Emergency medicine	0.59	0.089	3.95	0.591	
Internal medicine	4.44	0.568	34.84	0.155	
Trauma surgery	0.60	0	88.720	0.933	
Surgery, other	1.56	0.014	173	0.853	
General medicine	0.08	0.009	0.69	0.022	**
Not reported	1.79	0.135	23.7	0.66	
**Leading indication**					
Cardiovascular arrest	1				
Trauma	1.60	0.583	4.411	0.361	
others	0.36	0.104	1.275	0.114	

Sig, *p*-value; ***, *p* < 0.01; **, *p* < 0.05; *, *p* < 0.1; LL, lower limit; UL, upper limit.

## Data Availability

Data are available on request for academic purposes.
